# The Impact of Author Name Ambiguity on Bibliometric Validity. Comment on Ejaz et al. Bibliometric Analysis of Publications on the Omicron Variant from 2020 to 2022 in the Scopus Database Using R and VOSviewer. *Int. J. Environ. Res. Public Health* 2022, *19*, 12407

**DOI:** 10.3390/ijerph23050581

**Published:** 2026-04-30

**Authors:** Zineb Rhazzar, Khalid Ennibi, Nadia Touil

**Affiliations:** 1 Biomedical and Epidemiology Research Unit (URBE), Center of Virology, Infectious and Tropical Diseases, Mohammed V Military Training Hospital, Rabat 10000, Morocco; kennibi@yahoo.fr (K.E.); ntouil2003@gmail.com (N.T.); 2 Molecular Virology and Onco-Biology, Faculty of Medicine and Pharmacy, University Mohammed V, Rabat 10100, Morocco; 3 Mohammed VI Center of Research and Innovation (CM6RI), Mohammed VI University of Sciences and Health, Rabat 10100, Morocco

Ejaz et al. (2022) conducted a comprehensive bibliometric analysis of scientific publications on the Omicron variant of SARS-CoV-2, between 2020 and 2022 [1]. Drawing data from the Scopus database and using the Bibliometrix R package (Biblioshiny interface, RStudio, Boston, MA, USA) and VOSviewer (Leiden University, Leiden, The Netherlands) tools, the authors provided a timely overview of the research landscape during a critical stage of the COVID-19 pandemic. Their study effectively mapped publication trends, authorship patterns, and collaborative networks, revealing rapid scientific mobilization in response to the emergence of Omicron. Among their findings, the authors identified *Wang Y.* as the most productive author with 34 publications, followed by *Zhang Y.* and *Li J.*, reported as leading contributors in terms of publication count and citation frequency (Figure 6) [1].

However, while the analysis offers an informative overview of research activity, a critical methodological limitation appears to have been overlooked, namely, the ambiguity in author identification that arises during bibliometric data processing. The harmonization of author names process can lead to the aggregation of distinct individuals sharing the same surname and initials under a single author label. This phenomenon is particularly pronounced among common East Asian surnames such as *Wang*, *Zhang* and *Li*. These names appear with very high frequency, and when combined with identical initials (*Wang Y.*, *Zhang Y.*, and *Li J.*), they become statistically indistinguishable without additional identifiers. As a result, the entity labeled “*Wang Y.*” in bibliometric datasets often corresponds to multiple distinct researchers, for instance, *Wang Ying*, *Wang Yu* or *Wang Youchun*. Consequently, the apparent productivity and citation impact attributed to this single name may in fact represent the aggregated output of several individuals, thereby overestimating the influence of one author while simultaneously underrepresenting others.

Several internal contradictions and statistical improbabilities within the published data further substantiate this interpretation. First, a clear temporal implausibility concerns the individual output attributed to *Wang Y.* First discovered in South Africa and Botswana, the Omicron variant was announced as a Variant of Concern on 24 November 2021, and the dataset for the study was finalized on 1 July 2022 [1]. This leaves a narrow window of approximately seven months for the bulk of Omicron-specific research to have been conceived, conducted, peer-reviewed, and published. For a single researcher to produce 34 original articles within this timeframe, whether as a first author or co-author, averaging nearly five publications per month, is statistically highly improbable in high-impact biomedical research. This figure is, however, entirely consistent with the cumulative output of dozens of distinct researchers sharing the *Wang Y.* name pattern across Chinese academic institutions.

Second, a major discrepancy exists between reported productivity and citation impact. While *Wang Y.* is ranked as the most relevant author by document count, this name is entirely absent from the top ten globally cited authors reported in this study. Similarly, the most influential researchers identified by citation metrics, such as *Garcia-Beltran W.F.* (210 citations) and *Andrews N.* (165 citations), do not appear in the top productivity rankings [1]. This systematic disconnect between high document count and low citation impact strongly suggests that the apparent productivity of *Wang Y.* is a statistical artifact resulting from the merging of many authors with individually low citation counts, rather than the output of a single world-leading expert.

While direct access to the original dataset of Ejaz et al. (2022) is unavailable for individual-level verification, the consistent reappearance of *Wang Y.*, *Zhang Y.*, and *Li J.* as top-ranked authors across multiple independent bibliometric analyses [2,3] points to a reproducible methodological artifact. This pattern lends support to the interpretation that these names function as aggregated author clusters rather than single individuals. This limitation has been widely documented in the bibliometric literature, with Kim & Diesner (2016) demonstrating the distortive effects of initial-based name disambiguation on co-authorship network measurements of large-scale co-authorship networks, leading to inflated author productivity and erroneous network metrics [4]. Additionally, according to Xu & Hu [5], approximately 85% of the population of China, exceeding 1.1 billion people, shares only 129 surnames. The top five most frequent names alone represent 30.8% of the registered national population, with individual surnames reaching staggering absolute numbers: 108 million *Wangs*, 105 million *Lis*, 98 million *Zhangs*, 75 million *Chens* and 74 million *Lius*. This phenomenon is even more pronounced in South Korea, where nearly half of the population shares just three surnames (*Kim*, *Lee* and *Park*), reaffirming that author name ambiguity remains a pervasive challenge, and can significantly bias assessments of individual contributions and bibliometric indicators, even when multiple disambiguation methods are applied [5]. Although this problem is particularly acute with East Asian names due to the high prevalence of common names, it is not regionally confined. Any common surname–initial combination can be affected, such as *Smith* in Western contexts.

This issue is not unique to studies relying on a single data source, such as Ejaz et al. (2022) [1]. In our own bibliometric analysis on SARS-CoV-2 viral culture research (Rhazzar et al., manuscript under review), conducted across Scopus, Web of Science, and PubMed, we observed similar results with *Wang Y.*, *Zhang Y.* and *Li J.* appearing as the most prolific authors. However, upon manual verification of the publications attributed to these names, it became evident that these entries corresponded not to single individuals but rather to multiple distinct researchers sharing the same name pattern. To further clarify this ambiguity, Table 1 presents the results of a manual disambiguation performed for the abbreviated author label “*Wang Y.*”, illustrating the top 10 distinct authors sharing this same name pattern along with their number of articles. Notably, the label “*Wang Y.*” appeared as a single entity with 164 publications in the bibliometric analysis results; however, the manual disambiguation reveals that this publication output is, in fact, distributed among several different researchers. This strongly suggests that the apparent dominance of these names in bibliometric rankings is largely an artifact of name ambiguity rather than an accurate reflection of individual productivity.

Interestingly, this ambiguity does not necessarily originate exclusively from missing information in the bibliographic databases themselves. In my case, the bibliographic files extracted from Scopus, Web of Science and PubMed did contain the full author names. Even when importing these files into Bibliometrix (via Biblioshiny), and explicitly selecting the “Full names (if available)” option to preserve complete author information, the same problem persisted, with names still aggregated as *Wang Y.*, *Zhang Y.* and *Li J.* during the analysis. This observation raises the possibility that the simplification or truncation of author names may occur within the internal parsing and standardization algorithms of Bibliometrix, and not during data export from databases.

The broader implications of this ambiguity extend beyond simple productivity rankings. In the study’s Sankey diagram (Figure 11) [1], also referred to as a three-field plot, the interpretation of intellectual contributions may be affected by author name ambiguity, given that the visualization is based on aggregated relationships attributed to standardized text strings rather than unique individual identities. Therefore, the mis-aggregation results in artificially tall rectangles presenting names such as *Wang Y.* and *Zhang Y.*, and thickened flow links, creating a “super-node” that appears as a dominant global hub connecting unrelated countries and research clusters. Ultimately, the intellectual map misrepresents the actual structure of the field’s collaborative and thematic organization.

To address this limitation and enhance methodological rigor and transparency, future bibliometric studies should implement a multi-layered disambiguation strategy:

1. Manual curation of top-ranked authors by cross-checking their publication lists, their institutional affiliations, and, where accessible, email addresses or laboratory affiliations listed in the original publications.
2. ORCID-based disambiguation, which provides persistent author identities independent of name format [5]. However, it must be acknowledged that ORCID coverage remains uneven, with significant gaps for scientists in low- and middle-income countries who may not yet consistently use these identifiers. Relying solely on ORCID could therefore lead to the exclusion of important contributors, and it should be employed as a complementary rather than exclusive tool.
3. Algorithmic disambiguation, using machine learning approaches that cluster author records based on co-authorship patterns, shared keywords, institutional affiliations, and email domains, should be adopted as a standard preprocessing step within the Bibliometrix workflow prior to any network or productivity analysis.
4. Explicit methodological acknowledgement of the potential influence of name ambiguity on the reported rankings, as a minimum standard of transparency in bibliometric reporting.

The absence of any discussion of this issue in Ejaz et al. (2022) [1] suggests that the authors may not have been aware of this systematic bias. By systematically addressing these points, future studies can improve the reliability and interpretability of bibliometric indicators, particularly in global research contexts where naming conventions and data standardization practices vary widely.

## Figures and Tables

**Table 1:** Manual disambiguation of authors grouped under the name “*Wang Y.*”, showing the distribution of publications among distinct individuals.

Rank	Author Full Names	Number of Articles
1	*Wang Youchun*	25
2	*Wang Yanqun*	8
3	*Wang Ying*	7
3	*Wang Yining*	7
4	*Wang Yang*	5
4	*Wang Yixin*	5
4	*Wang Yunfei*	5
5	*Wang Yan*	4
5	*Wang Yao*	4
5	*Wang Yu*	4

## References

[1] Ejaz H., Zeeshan H.M., Ahmad F., Bukhari S.N.A., Anwar N., Alanazi A., Sadiq A., Junaid K., Atif M., Abosalif K.O.A. (2022). Bibliometric Analysis of Publications on the Omicron Variant from 2020 to 2022 in the Scopus Database Using R and VOSviewer. Int. J. Environ. Res. Public Health.

[2] Chen Y., Cao B., Zhou Q., Liu Y., He Q., Zhao M. (2023). Bibliometric Evaluation of 2020–2022 Publications on COVID-19-Related Cardiovascular Disease. Front. Cardiovasc. Med..

[3] Qamar A.M., Khan R.U., Alsuhibany S.A. (2021). Large-Scale Bibliometric Analysis of Coronavirus. Int. J. Des. Nat. Ecodyn..

[4] Kim J., Diesner J. (2016). Distortive Effects of Initial-Based Name Disambiguation on Measurements of Large-Scale Coauthorship Networks. J. Assoc. Inf. Sci. Technol..

[5] Xu S.B., Hu G. (2025). Rethinking the Author Name Ambiguity Problem and beyond: The Case of the Chinese Context. Account. Res..

